# A Cysteine-Reloading Process Initiating the Biosynthesis of the Bicyclic Scaffold of Dithiolopyrrolones

**DOI:** 10.3390/antibiotics12040787

**Published:** 2023-04-20

**Authors:** Yan Chen, Yanqin Tu, Tingyu Pan, Zixin Deng, Lian Duan

**Affiliations:** Key Laboratory of Combinatory Biosynthesis and Drug Discovery (Ministry of Education), School of Pharmaceutical Sciences, Wuhan University, Wuhan 430071, China; chen-yan@whu.edu.cn (Y.C.); t18211733145@163.com (Y.T.); pan_tingyu@whu.edu.cn (T.P.); zxdeng@sjtu.edu.cn (Z.D.)

**Keywords:** dithiolopyrrolone, non-ribosomal peptide synthase, thiolutin, adenylation domain

## Abstract

Dithiolopyrrolone antibiotics are well known for their outstanding biological activities, and their biosynthesis has been studied vigorously. However, the biosynthesis mechanism of the characteristic bicyclic scaffold is still unknown after years of research. To uncover this mechanism, a multi-domain non-ribosomal peptide synthase DtpB from the biosynthetic gene cluster of thiolutin was selected as an object to study. We discovered that its adenylation domain not only recognized and adenylated cysteine, but also played an essential role in the formation of the peptide bond. Notably, an eight-membered ring compound was also discovered as an intermediate during the formation of the bicyclic structure. Based on these findings, we propose a new mechanism for the biosynthesis of the bicyclic scaffold of dithiolopyrrolones, and unveil additional functions of the adenylation domain.

## 1. Introduction

**Figure 1:**
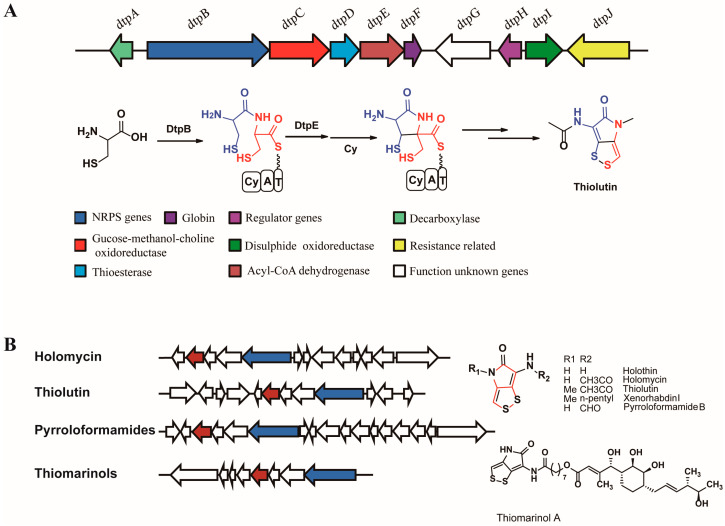
Biosynthetic gene clusters and putative pathway of dithiolopyrrolones. (**A**) Putative biosynthetic pathway of thiolutin. (**B**) Biosynthetic gene clusters of reported dithiolopyrrolones. The genes coding for acyl-CoA dehydrogenase are in red and the genes coding for NRPS are in blue.

Dithiolopyrrolone antibiotics have been studied for years [1,2,3] due to their broad-spectrum anti-bacterial activity and anti-cancer property [4,5,6,7]. The mechanism of their biosynthesis has always been an intriguing research topic for scientists to explore their clinical utilization [5]. Since the gene cluster directing holomycin biosynthesis in *Streptomyces clavuligerus* has been reported by Li et al. (2010) [8] and Huang et al. (2011) [9] (Figure 1A), several other biosynthetic gene clusters of dithiolopyrrolones were identified [10]. A similar report identified a gene cluster associated with thiolutin biosynthesis in *Saccharothrix algeriensis* NRRL B-24137 [11]. Another report provided detailed biochemical information on the biosynthesis of a “hybrid” molecule, thiomarinol, which suggested a new idea for expanding dithiolopyrrolone diversity [12]. Genome mining of the conserved genes from the reported biosynthetic gene clusters of dithiolopyrrolones revealed pyrroloformamides with a modification at the disulfide bond rather than an amide nitrogen [13]. Although many gene clusters have been identified, understanding of the biosynthetic mechanism of dithiolopyrrolones is still limited [14], particularly the assembly of the bicyclic pyrrolinodithiole scaffold.

According to reported gene clusters, the skeleton of pyrrolinodithiole is putatively based on a five-membered lactam. A non-ribosomal peptide synthase (NRPS) and a putative acyl-CoA dehydrogenase are responsible for the biosynthesis of five-membered lactam (Figure 1B). The corresponding NRPS protein consists of a cyclization domain (Cy), an adenylation domain (A), and a peptidyl carrier protein domain (PCP). According to a predicted mechanism for dithiolopyrrolone biosynthesis, the Cy domain catalyzes a condensation reaction between two activated cysteine molecules to generate a peptide bond. However, to prove this hypothesis, there is no direct evidence other than the presence of free dipeptides in the solution [8]. In this study, we chose DtpB protein (referred to as NRPS in thiolutin biosynthetic gene cluster) to study the reaction between two cysteine molecules on the PCP domain and suggested a new mechanism for the biosynthesis of the bicyclic scaffold.

## 2. Results

### 2.1. Discovery of a Novel Intermediate Derived from Cysteine Led to a Proposed Mechanism for the Cysteine Reloading Process

**Figure 2:**
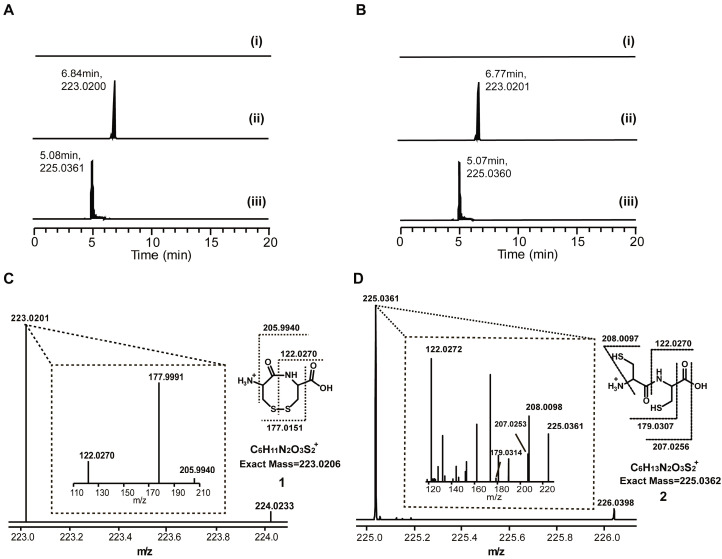
Special bicyclic structure of dithiolopyrrolones and analysis of products from cysteine loading assays with DtpB and DtpB-APCP: (**A**) Molecular weight extraction on MS (mass spectrometry) spectrum of hydrolysis products of DtpB; (i) control without ATP, (ii) reaction assay, (iii) reaction assay with TCEP. (**B**) Molecular weight extraction on MS (mass spectrometry) spectrum of hydrolysis products of and DtpB-APCP. (**C**) MS^2^ spectrum of compound **1**. (**D**) MS^2^ spectrum of compound **2**.

To study functions of the Cy and A domains, two purified proteins, full DtpB and the Cy domain omitting DtpB (DtpB-APCP), were incubated with cysteine. In matrix-assisted laser desorption/ionization-time of flight (MALDI-TOF) results, single-cysteine-loaded proteins were observed as high-intensity signals for both assays performed with DtpB and DtpB-APCP (Figures S1–S3). However, signals such as dipeptide-loaded proteins might have been buried, possibly due to their low intensity. Furthermore, a hydrolysis reaction was introduced to release all the compounds grabbed by the PCP domain. A compound with a molecular weight (MW) of approximately 223 Da was found in both assays with DtpB (Figure 2A) and DtpB-APCP (Figure 2B). The MS^2^ spectrum suggested that this compound (compound **1**, observed MS = 223.0201 Da, calculated MS = 223.0206 Da) (Figure 2C) was derived from the Cys–Cys dipeptide with an additional disulfide bond that was oxidized from sulfhydryl groups of cysteine molecules. To further verify compound **1**, a reducing agent TCEP (tris (2-carboxyethyl) phosphine), that can break the disulfide bond, was added after incubation. As a result, another compound with an approximate MW of 225 Da was observed while the peak of compound **1** disappeared (Figure 2A,B). The MS^2^ spectrum of this compound (compound **2**, observed MS = 225.0351 Da, calculated MS = 225.0352 Da) (Figure 2D) suggested it was a Cys–Cys dipeptide. The shift of 2 Da in the MW of these 2 compounds was caused by the reduction of compound **1** by TCEP. This observation supports the presence of a disulfide bond in compound **1** and further confirms its structure. In addition to discovering compound **1**, the results also provide an interesting presumption that the formation of peptides from dipeptide precursors of dithiolopyrrolones solely relies on the A and PCP domains of NRPS.

**Figure 3:**
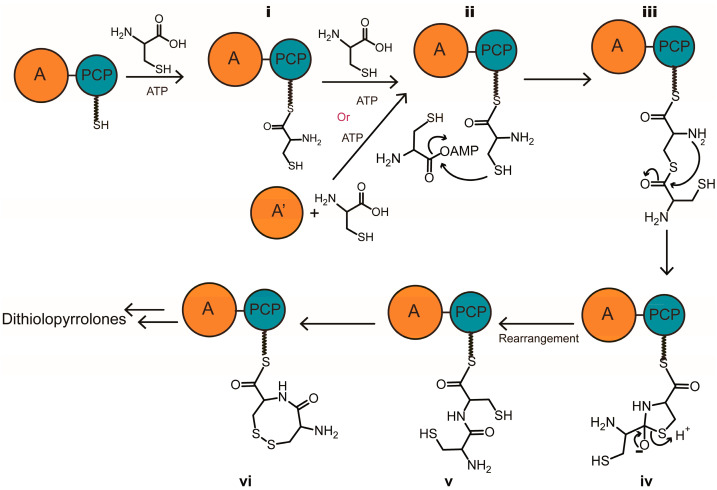
A proposed mechanism for the cysteine reloading process catalyzed by A domain.

A domain itself that assists the condensation of peptide bond has been reported by the Kobayashi group [15,16]. In their research, dipeptides can be generated between cysteine and another amino acid when this amino acid was adenylated by the relative A domain. There were two main steps included in the mechanism of this reaction. Firstly, the sulfhydryl of cysteine performed a nucleophilic attack on the adenylated amino acid to generate a thioester. Then, this thioester bond is rearranged into a peptide bond to generate the dipeptide. Considering this rearrangement mechanism and compound **1** discovered above, we envisaged a proposed mechanism for the formation of compound **1** (Figure 3). At the beginning of this mechanism, the first molecule of cysteine was adenylated and loaded onto the PCP domain in the normal way. The second molecule of cysteine, which was adenylated by the same A domain or a different one, bonded to the first one by forming a thioester. Then, a rearrangement occurred to transfer the thioester into a dipeptide. At the end of this reaction, a disulfide bond formed between these two cysteine molecules to give an eight-member ring product, which was discovered as compound **1**.

### 2.2. Experiments to Investigate and Correct the Proposed Mechanism

**Figure 4:**
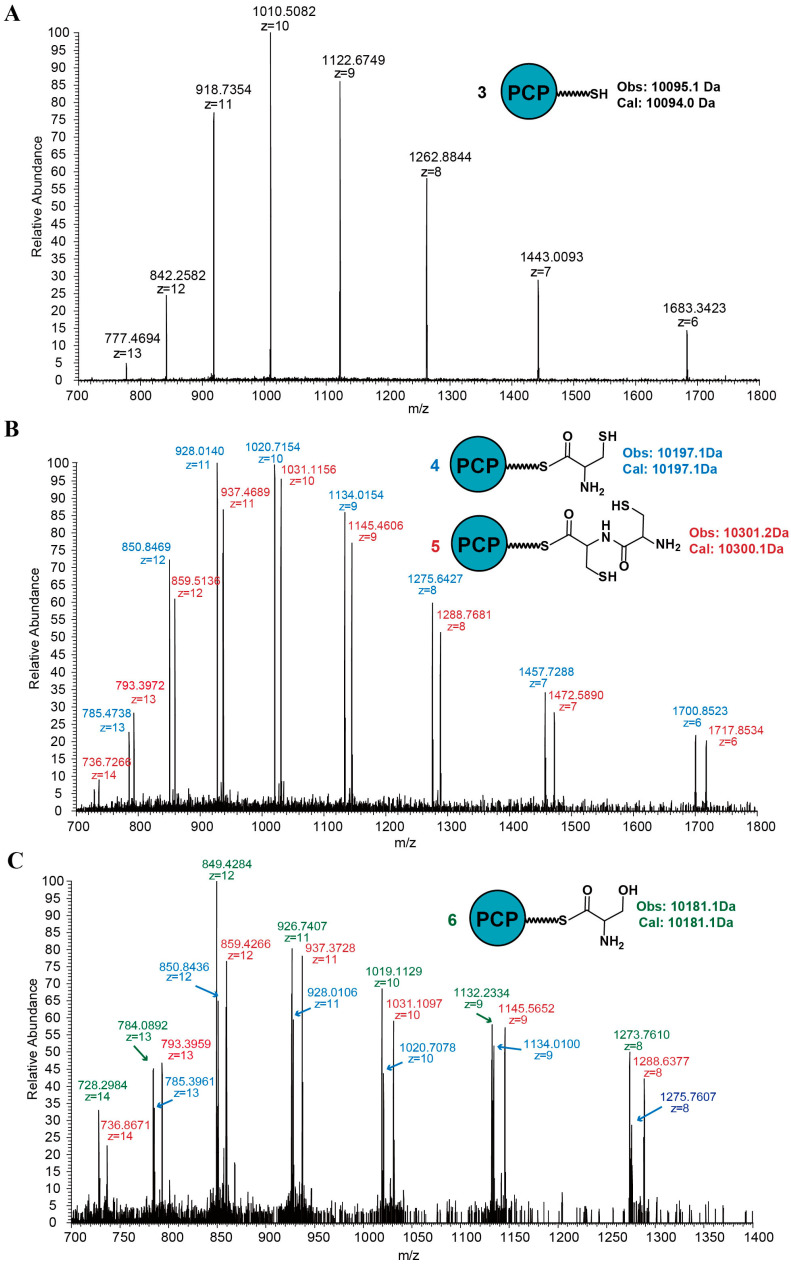
MS spectrum of products from amino acid loading assays with DtpB-PCP after TEV digestion. MS mass/charge (*m*/*z*) envelopes and mass deconvolution (Obs.) and expected mass (Exp.) are shown. (**A**) MS spectrum of *holo*-PCP domain from cysteine loading assay. (**B**) MS spectrum of single-cysteine-loaded PCP domain and Cys–Cys dipeptide-loaded PCP domain from cysteine loading assay. (**C**) MS spectrum of three different products from mixed substrates loading assay. Single-cysteine-loaded PCP domain, Cys–Cys dipeptide-loaded PCP domain, and single-serine-loaded PCP domain were observed.

To test our proposal, a TEV recognition site was introduced into DtpB-APCP between the A domain and PCP domain. Based on the TEV recognition site, the PCP domain with different small molecules loaded could be observed clearly using LC-HRMS after TEV digestion and TCEP treatment (TCEP treatment was needed for screening the high signal of side production which was formed by disulfide bond connection between Cys and *holo*-PCP domain). As a result, three PCP-domain-associated proteins were observed from extraction of the incubation assays, a *holo*-PCP domain (compound **3**, observed MS = 10,095.1 Da, calculated MS = 10,094.0 Da) (Figure 4A), a single-cysteine-loaded PCP domain (compound **4**, observed MS = 10,197.1 Da, calculated MS = 10,197.1 Da), and a Cys–Cys dipeptide-loaded PCP domain (compound **5**, observed MS = 10,301.2 Da, calculated MS = 10,300.1 Da) (Figure 4B). The discovery of compound **4** and compound **5** proved the conclusion that the A-PCP bidomain catalyzed the formation of peptide bond of intermediate v in the proposed mechanism directly.

Based on the experimental method established above to observe the products loaded on the PCP domain, further studies on the relationship between the A domain and the second molecule of cysteine were carried out. In the first experiment, the PCP domain was separated from the A domain and regarded as an intermolecular state compared to the intramolecular state at which the PCP domain was linked to the A domain. The individual A domain, and apo-DtpB and DtpB-CyA bidomain were used in the incubation reaction, and all the results showed that only single-cysteine-loaded product compound **4** was observed (Figure S4). The different results between intramolecular state and intermolecular state suggested that compound **5** can only be produced when the PCP domain was linked to the A domain. According to this result, an undetermined step in the previously proposed mechanism was clear. It was that the second adenylated cysteine molecule could only be provided by the same A domain as the first one.

In another experiment to study the A domain, cysteine and serine were added successively in the reactions as substrates with DtpB-APCP or DtpB-A. For the reaction with the individual A domain, in addition to Cys–Cys dipeptide, Ser–Cys dipeptide was also discovered as one of the products (Figure S5). For the reaction with DtpB-APCP bidomain, to avoid the affection of the preference of the A domain, serine was added after excessive cysteine was cleaned up. From the result, only compound **5** was observed besides the single-cysteine-loaded PCP domain compound **4** and single-serine-loaded PCP domain compound **6** (observed MS = 10,181.1 Da, calculated MS = 10,181.1 Da) (Figure 4C). According to the proposal, the A domain adenylated both serine and cysteine in this reaction; the Ser–Cys dipeptide was supposed to be discovered as one of the products when mixed substrates were added. However, as the results showed, Ser–Cys dipeptide can only be discovered in the reaction with the individual A domain, not in the reaction with A-PCP bidomain. These results implied that condensation reaction catalyzed by bidomain may have an additional mechanism to limit the substrate to cysteine.

**Figure 5:**
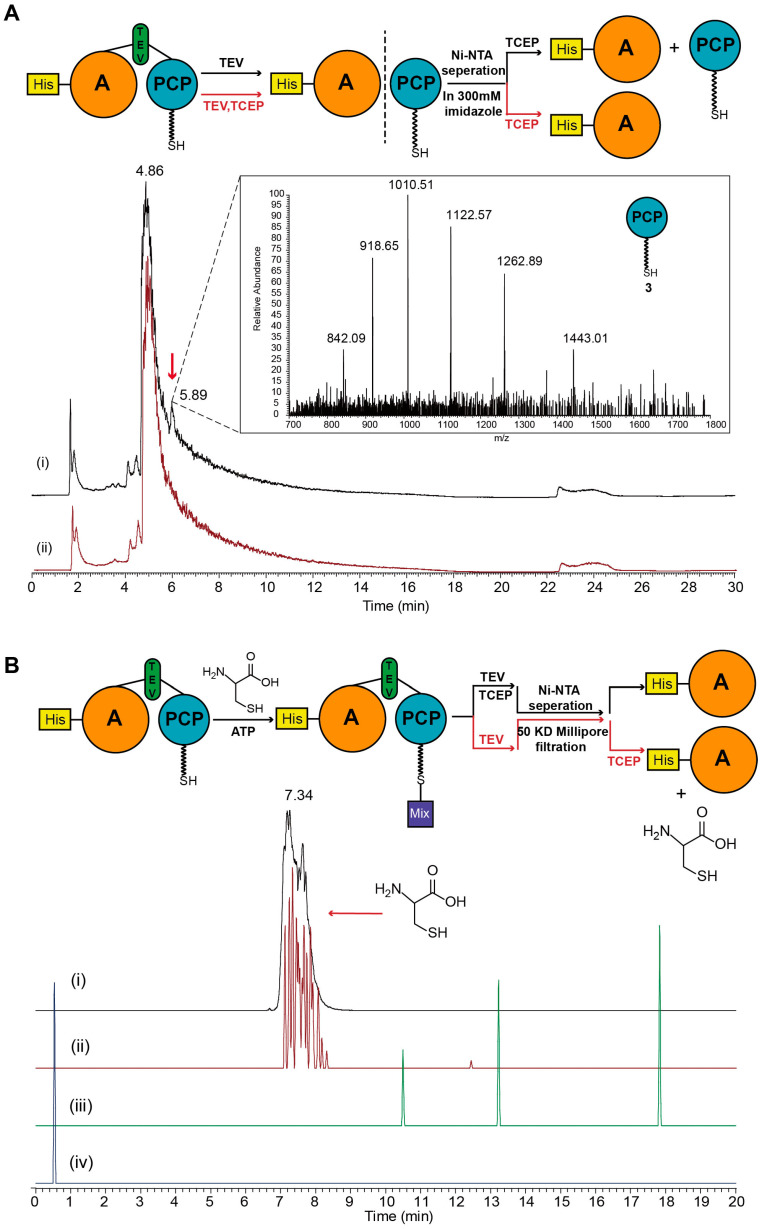
Experiments to discuss the function of A domain. (**A**) The description of incubation reactions without cysteine. (i) TIC of the assay in which TCEP was added before detection. (ii) TIC of the assay in which TCEP was added at the same time as TEV. The peak at 5.89 min was identified as compound **3**. (**B**) The description of incubation reactions which were set out to verify the additional function of A domain. (i) MW extraction for exact MW of cysteine from TIC of a standard sample of cysteine. (ii) MW extraction for exact MW of cysteine from TIC of the assay to which TCEP was added before detection. (iii) MW extraction for exact MW of cysteine from TIC of the assay in which TCEP was added before Ni-NTA separation. (iv) MW extraction for exact MW of cysteine from TIC of the assay in which mutated protein DtpB-APCP-C168A was used and TCEP was added before detection.

Keeping this additional mechanism in mind, an interesting discovery revealed the tip of the iceberg of the reaction mechanism during the digestion of proteins containing TEV sites. In the incubation reaction, if cysteine had not been added during the whole process, and TCEP only added before the final detection, compound **3** could be detected both by LC-HRMS (Figure 5A) and SDS-PAGE (Figure S6) in the 300 mM imidazole washing part of the Ni-NTA separation. As the *holo*-PCP domain without His-tag was not supposed to be grabbed by Ni-NTA, there should be an additional connection between the A domain and PCP domain besides the TEV site to retain the PCP domain until 300mM imidazole was used. In contrast, the addition of TCEP, which was usually used to break disulfide bond during the TEV digestion, vanished this connection. According to this, a disulfide bond may be formed between the A domain and the PCP domain occasionally, which resulted in the retention of the PCP domain on the Ni affinity column.

To determine the cysteine residue involved in disulfide bond formation on the A domain, a multiple-sequences alignment was set out among A domains involved in biosynthesis of dithiolopyrrolones; A domains which followed Kobayashi’s rearrangement mechanism [17] and regular A domains recognized cysteine as a substrate [18,19] (Figure S7). In addition to 10 reported conserved regions [20,21,22,23], A domains from dithiolopyrrolone biosynthetic pathways had a specially conserved cysteine about 15 amino acids behind the second conserved region compared to alanine in other A domains. Site mutations using alanine, serine, or methionine to replace this cysteine produced corresponding mutated DtpB and DtpB-APCP. Cysteine loading assays with these mutants showed that the mutation on conserved Cys site abolished the formation of dipeptide products, so that only the single-cysteine-mounted PCP could be detected (Figure S8). These results suggested that this conserved cysteine was essential for loading the second molecule of cysteine. 

To confirm this idea, we designed additional experiments focusing on the A domain. Unlike previous experiments, the A domain instead of the PCP domain was isolated and cleaned from each assay after TEV digestion, then treated with TCEP before extraction. As shown on the LC-HRMS spectrums (Figure 5B), cysteine was discovered in the extraction of the assay that DtpB-APCP was used but not in the assay that mutant DtpB-APCP-C168A was used. These results suggested that the A domain could save a cysteine molecule by forming a disulfide bond with the conserved cysteine residue. According to the results of incubation reactions of mutants, this saved molecule of cysteine could be used as the second molecule of cysteine which was supposed to be used to form a dipeptide in the proposal for the mechanism of the biosynthesis of compound **1**.

## 3. Discussion

**Figure 6:**
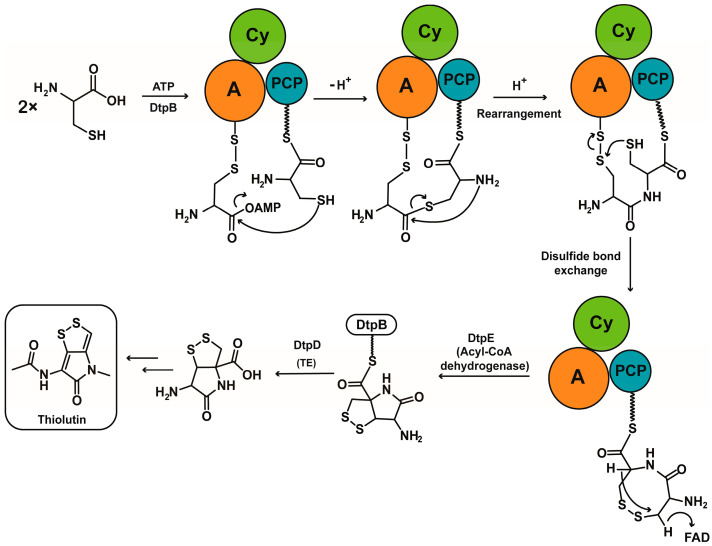
Proposed mechanism for the biosynthesis of bicyclic pyrrolinodithiole scaffold of thiolutin.

Based on all the results, a more detailed mechanism for the biosynthesis of the bicyclic pyrrolinodithiole scaffold of thiolutin was proposed (Figure 6). In this mechanism, two molecules of cysteine were grabbed by DtpB first. One molecule of cysteine was adenylated and loaded onto the PCP domain, and the other one was linked to the A domain by a disulfide bond and also adenylated by the A domain. After that, a thioester bond formed between these two molecules of cysteine, and a rearrangement occurred subsequently to transfer the thioester bond into a peptide bond. The next step was a disulfide bond exchange between the free sulfhydryl group of the cysteine loaded on the PCP domain and the disulfide bond on the A domain. As a result, compound **1** with an eight-membered ring was produced and linked to PCP domain. Because of the special conformation of an eight-membered ring, DtpE, which was annotated as acetyl-CoA dehydrogenase, was proposed to catalyze the dehydrogenation between C1 and C5 of compound **1** with the assistance of the Cy domain to directly generate the bicyclic scaffold. Finally, in the presence of various post-modified proteins after TE hydrolysis, the biosynthesis of thiolutin was completed. 

According to this mechanism, the puzzling results in the mixed substrates feeding experiments can also be explained. As one of the substrates, serine was not only unable to attach to the cysteine residue in the A domain as an acceptor of the sulfhydryl group, but was also inadequate as a donor of the sulfhydryl group. As a result, the thioester intermediate that was important for this mechanism cannot be formed, and thus the related dipeptide derivatives cannot be produced. 

## 4. Conclusions

The function of NRPS which determines the basic bicyclic skeleton is one of the missing pieces of the biosynthesis pathway of dithiolopyrrolones. These NRPSs all contain a cyclization domain, a protein carrier domain, and a normal adenylation domain which is considered to be responsible for recognizing and activating specific amino acids. In our study, this A domain is proved to be essential for creating a Cys–Cys dipeptide derived product, while no condensation domain is needed. A revised mechanism for the biosynthesis of bicyclic pyrrolinodithiole scaffold is also proposed. In this mechanism, the A domain and PCP domain each carry a molecule of cysteine in different ways to complete the synthesis of the dipeptide. Additionally, a new intermediate compound **1** with an eight-member ring and disulfide bond is discovered, which leads to a new pathway for the formation of the bicyclic scaffold and post-modification. As disulfide bonds in dithiolopyrrolones are a self-protective mechanism that limit the activity of these compounds and protect the producing strain [7], the presence of disulfide bond at the very beginning of the biosynthesis of dithiolopyrrolones indicates that this protection is maintained throughout the biosynthesis process, providing double insurance with HlmI, which is responsible for forming a disulfide bond at the last step of the biosynthesis of holomycin [24]. The role of the remaining acyl-CoA dehydrogenase encoded by DtpE in the biosynthetic gene cluster of thiolutin has not been confirmed, as it is difficult to observe the MW change caused by dehydrogenase on PCP domain. Ultimately, we anticipate that the results gained in this study will help people to understand the assembly logic of the bicyclic ring of dithiolopyrrolones and expand knowledge on A domains with special functions.

## 5. Materials and Methods

*Escherichia coli* competent cells were purchased from Shanghai Weidi Biotechnology Co., Ltd. (Shanghai, China). The restriction enzymes used were purchased from Thermo Fisher Scientific—CN Inc. (Shanghai, China). IPTG, ATP, L- cysteine, L-glycine, L-methionine, L-threonine, and L-serine were purchased from Sigma-Aldrich (Shanghai, China) Trading Co., Ltd. (Shanghai, China).

### 5.1. Cloning and Expression of DtpB and DtpB-APCP

Details of used primers are listed in Table S1. The DNA fragments *dtpB* and *dtpB*-APCP were amplified by PCR using cosmid 16F5 as a template. To produce N-terminally His6-tagged proteins, the expression plasmid was prepared by ligation of pET-28a (+) digested by Nde I and Xho I and purified DNA fragments. The recombinant plasmids were verified by digestion and DNA sequencing.

*E. coli* BL21 (DE3) cells carrying plasmid DNA were grown in 2 L LB containing kanamycin (50 μg/mL) at 37 °C. The incubation temperature was reduced to 18 °C until OD600 reached 0.6–0.8, and then 0.5 mM isopropyl β-D-thiogalactoside (IPTG) was added. After 24 h of culture, the cells were harvested using centrifugation (4500× *g*, 15 min), washed twice, and then suspended in buffer A (20 mM Tris-HCl, 300 mM NaCl, 25 mM imidazole, 10% glycerol, pH 8.0). The resuspended cells were lysed using a homogenizer (EmulsiFlex-C3, Avestin Inc., Ottawa, ON, Canada), and the cell debris was removed by centrifugation (12,000× *g*, 30 min). The supernatant was loaded onto a nickel-chelating column His-Trap HP (5 mL) (GE Life Science, Zhejiang, China) using a peristaltic pump (Longer Pump BT100-1L, Baoding Longer Precision Pump Co., Ltd., Baoding, China). The column was washed with buffer A and protein eluted with buffer B (20 mM Tris-HCl, 300 mM NaCl, 300 mM imidazole, 10% glycerol, pH 8.0). The eluted protein was collected and concentrated to 2.5 mL with a 15 mL Amicon (Millipore, Burlington, MA, USA) with the appropriate molecular weight cut-off. The concentrated protein was transferred into store buffer (20 mM Tris-HCl, 100 mM NaCl, 10% glycerol, pH 8.0) by using a PD-10 desalting column (GEHealthcare Bio-Sciences Corp., Newark, NJ, USA) or further purified by size exclusion chromatography (HiloadTM 16/600 SuperdexTM 200 pg, GE Healthcare Bio-Sciences AB, Uppsala, Sweden)) using store buffer. Protein samples without imidazole were concentrated and stored at −80 °C. The phosphopantetheinylated protein was expressed and purified from the *E. coli* BAP-1 strain following the same protocols as described above. The purified proteins were analyzed using SDS-PAGE.

### 5.2. Cysteine Loading Assays with DtpB or DtpB-APCP

The function of the adenylation domain in the DtpB or DtpB-APCP was tested. *holo*-DtpB (20 µM) was mixed with 5 mM Cys, 5 mM ATP, 5 mM MgCl_2_, and 50 mM HEPES (pH 8.0) to a final volume of 500 mL. The reaction was incubated for 2 h at 30 °C. Proteins were desalted and concentrated to ~10 mg/mL with a 0.5 mL centrifugal filter (100 K nominal molecular weight limit, Amicon^®^ Ultra, Merk Millipore Ltd., Cork, Ireland); *holo*-DtpB-APCP was tested in the same way. The control groups for DtpB and DtpB-APCP were treated in the same way without ATP. Matrix-assisted laser desorption/ionization time-of-flight mass spectrometry (AB SCIEX TOF/TOF 5800, AB Sciex LLC, Applied Biosystems, Foster City, CA, USA) was used to determine the exact MW of proteins.

After analysis using MALDI-TOF, samples were hydrolyzed by alkali individually. Proteins were precipitated with an equal volume of 20% trichloroacetic acid, and the protein precipitations were washed with 10% trichloroacetic acid twice. The proteins were hydrolyzed by adding 100 µL 0.1 M NaOH and the samples were incubated at 65 °C for 5 min. Then, proteins were precipitated again with 5 µL 50% trifluoroacetic acid. After centrifugation, supernatants were injected into a C18 column (5 Å, 100 × 4.6 mm, Phenomenex Inc., Torrance, CA, USA) operating at 0.8 mL/min flowrate for MS detection (Thermo Scientific LTQ XL Orbitrap mass spectrometer, Thermo Fisher Scientific Inc., Waltham, MA, USA). The mobile-phase solvents (A: water and B: MeCN) were supplemented with 0.1% (*v*/*v*) formic acid and elution profiles were shown in Table S2.

### 5.3. Cysteine Loading Assays with DtpB or DtpB-APCP

Because of the difficulty in detecting mass spectrometry of proteins with high MW and the considerably large error of MALDI-TOF, we inserted a protease cleavage site between the A domain and PCP domain. One of the most promising ideas was to find an effective protease that can recognize the cleavage site and cut off the PCP domain from a large protein.

To construct a plasmid of NRPS protein containing the TEV protease cleavage site at a specific position, we used the plasmid prepared to express the original protein as the template. We then obtained 5′ upstream fragment F1 and 3′ downstream fragment R1 by using primers containing a full sequence of the TEV recognition site. A new plasmid was generated by the recombination of the two fragments F1 and R1 through in-fusion cloning. Constructing both DtpB-APCP-TEV and DtpB-TEV followed the same protocols.

Expression and purification of the *holo* form fusion proteins were performed using the same steps described above. The TEV protease cleaved the harvested protein, and the ratio of protease to protein was 1:100. The TEV protease can effectively recognize the cleavage site and cut off the PCP domain completely in 1 h at room temperature.

*holo*-DtpB-TEV was digested with TEV protease at 4 °C overnight and the product was purified through a Ni-NTA column to obtain protein *holo*-DtpB-PCP and protein DtpB-CyA. DtpB-A was obtained by digesting *holo*-DtpB-APCP-TEV following the same protocols as described above.

### 5.4. Cys Loading Assays with PCP Domain and A Domain in Intermolecular Stage

*apo*-DtpB (10 mM) was incubated with *holo*-DtpB-PCP (100 µM) in the presence of 5 mM L-Cys, 5 mM ATP, 5 mM MgCl_2_, and 50 mM HEPES (pH 8.0). After incubation at 30 °C for 2 h, products were analyzed using LC-HRMS. After treatment with 5 mM TCEP, samples were injected into an ZORBAX 300SB-C8 (5 Å, 4.6 × 150 mm, Agilent Technologies Inc., Santa Clara, CA, USA) column operating at a 0.8 mL/min flow rate. Solvents (A: water and B: MeCN) were supplemented with 0.1% (*v*/*v*) formic acid, and the HPLC elution profile is shown in Table S3.

### 5.5. Cys Loading Assays Conjugated with TEV Digestion

To study the function of DtpB-TEV, *holo*-DtpB-TEV (20 µM) was mixed with 5 mM L-Cys, 5 mM ATP, 5 mM MgCl_2_, and 50 mM HEPES (pH 8.0) to a final volume of 500 mL. After incubation at 30 °C for 2 h, TEV protease was added and the reaction was incubated for a further 1 h at room temperature. The DtpB-PCP was purified through a Ni-NTA column and concentrated with a 0.5 mL centrifugal filter (3 K nominal molecular weight limit). After treatment with 5 mM TCEP, the samples were analyzed using MS. DtpB-APCP-TEV was tested as same as DtpB-TEV. TEV cleavage, purification, and LC-MS analysis were performed as described above.

### 5.6. Amino Acid Loading Assays with Mixed Substrates

To investigate the mechanism of Cys–Cys dipeptide formation without Cy domain, the amino acid substrate loading experiment was repeated with different amino acids. L-glycine, L-methionine, L-threonine, and L-serine were examined with apo-DtpB and *holo*-DtpB-PCP. The reaction mixture contained *apo*-DtpB (10 mM), *holo*-DtpB-PCP (100 mM), 5 mM amino acid substrate, 5 mM ATP, 5 mM MgCl_2_, and 50 mM HEPES (pH 8.0). These reactions were incubated at 30 °C for 2 h, then monitored by LC-MS after treatment with 5 mM TCEP.

DtpB-APCP-TEV (20 µM) was mixed with 5 mM L-Ser, 5 mM ATP, 5 mM MgCl_2_, and 50 mM HEPES (pH 8.0) to a final volume of 500 mL. After 2 h of incubation at 30 °C, the reaction was digested by TEV protease. The DtpB-PCP was purified and concentrated with a 0.5 mL centrifugal filter (3 K nominal molecular weight limit). After treatment with 5 mM TCEP, the samples were sent for MS analysis.

We anticipated that DtpB would synthesize Ser–Cys dipeptide in solution. Mixed substrates of L-Cys and L-Ser were examined with *apo*-DtpB. *apo*-DtpB (20 µM) was mixed with 0.5 mM L-Cys, 4.5 mM L-Ser, 5 mM ATP, 5 mM MgCl_2_, and 50 mM HEPES (pH 8.0) to a final volume of 100 mL. The mixture was quenched with an equal volume of methanol for MS analysis.

Mixed substrates of L-Cys and L-Ser were also incubated with *holo*-DtpB-APCP-TEV. *holo*-DtpB-APCP-TEV (20 µM) was mixed with 0.5 mM L-Cys, 4.5 mM L-Ser, 5 mM ATP, 5 mM MgCl_2_, and 50 mM HEPES (pH 8.0) to a final volume of 500 mL. The following steps are the same as DtpB.

### 5.7. Site-Directed Mutagenesis on DtpB-TEV and DtpB-APCP-TEV

We designed the site-directed mutations according to the amino acid sequences of DTPB-TEV and DTPB-APCP-TEV. The mutation sites of DTPB-TEV included C566A, C566S, and C566M, and the mutation sites of DTPB-APCP-TEV included C168A, C168S, and C168M. Corresponding primer pairs designed to construct site-directed mutants are listed in Table S4. After verifying the mutation sequence, the recombinant plasmid was transformed into the competent bacterial BAP1 for heterologous expression. Under the same conditions, protein expression, purification, and cysteine loading experiments were carried out following the same steps described above.

## Data Availability

The data presented in this study are available on request from the corresponding author. The data are not publicly available due to data security policy of Wuhan Univeristy.

## Supplementary Materials

The following supporting information can be downloaded at https://www.mdpi.com/article/10.3390/antibiotics12040787/s1, Figure S1: DNA electrophoresis of digested plasmids; Figure S2: Characterizations of the proteins by SDS-PAGE; Figure S3: MALDI-TOF results of L-Cys loading assays; Figure S4: Reaction results of DtpB-PCP with adenylation domains and cysteine at the intermolecular stage; Figure S5: MS spectrums of dipeptides produced by individual A domain *apo*-DtpB; Figure S6: Tricine-SDS-PAGE of DtpB-APCP after incubation without cysteine and TEV digestion; Figure S7: Multiple sequence alignment of adenylation domains; Figure S8: MS spectrum results of cysteine loading assays with site-mutated A-PCP bidomain of DtpB; Table S1: PCR primers used for protein expression; Table S2: HPLC elution profile for dipeptides; Table S3: HPLC elution profile for PCP domain; Table S4: PCR primers used for site-directed mutagenesis.
