# Supplementary material for: A Cysteine-Reloading Process Initiating the Biosynthesis of the Bicyclic Scaffold of Dithiolopyrrolones
Source: Antibiotics (Basel). 2023 Apr 20;12(4):787. doi: 10.3390/antibiotics12040787 (PMC10134970; doi:10.3390/antibiotics12040787)
Supplement: Supplementary file 1 [file antibiotics-12-00787-s001.zip › antibiotics-2345437-supplementary.pdf]

Supporting Information

A Cysteine-reloading Process Initiating the Biosynthesis of the Bicyclic Scaffold of Dithiolopyrrolones

Yan Chen[1], Yanqin Tu[1], Tingyu Pan[1], Zixin Deng[1] and Lian Duan[1,*]

Table of Contents

S1 Supporting figures

Figure S1. DNA electrophoresis of digested plasmids

Figure S2. Characterizations of the proteins by SDS-PAGE.

Figure S3. MALDI-TOF results of L-Cys loading assays.

Figure S4. Reaction results of DtpB-PCP with adenylation domains and cysteine at the intermolecular stage.

Figure S5. MS spectrums of dipeptides produced by individual A domain *apo*-DtpB.

Figure S6. Tricine-SDS-PAGE of DtpB-APCP after incubation without cysteine and TEV digestion.

Figure S7. Multiple sequence alignment of adenylation domains.

Figure S8. MS spectrum results of cysteine loading assays with site-mutated A-PCP bidomain of DtpB.

S2 Supporting tables

Table S1. PCR primers used for protein expression.

Table S2. HPLC elution profile for dipeptides.

Table S3. HPLC elution profile for PCP domain.

Table S4. PCR primers used for site-directed mutagenesis.

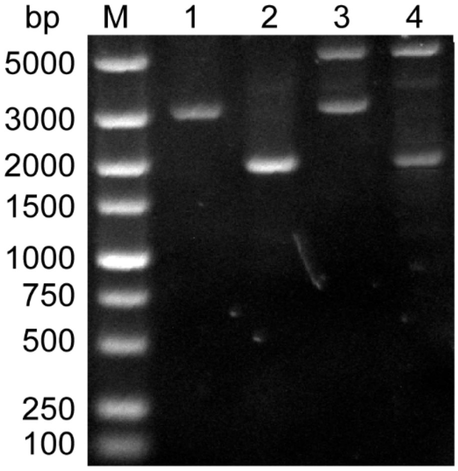

Figure S1. DNA electrophoresis of digested plasmids . M: molecular weight marker

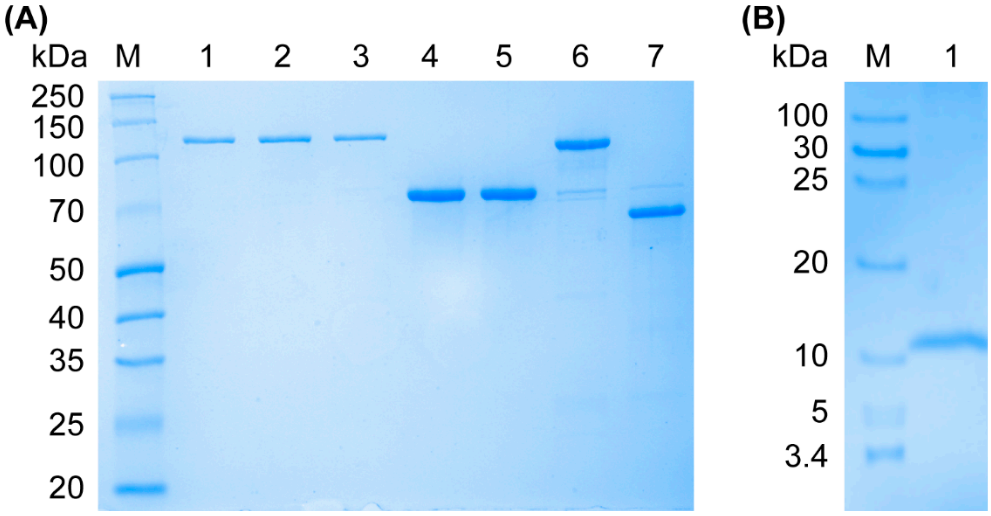

Figure S2. Characterization of the proteins by SDS-PAGE. All proteins contained an N-terminal His^6-tagged. SDS-PAGE analysis of apo-DtpB (line 1), holo-DtpB (line 2), holo-DtpB-TEV (line 3), holo-DtpB-APCP (line 4), holo-DtpB-APCP-TEV (line 5), DtpB-CyA (line 6), DtpB-A (line 7).

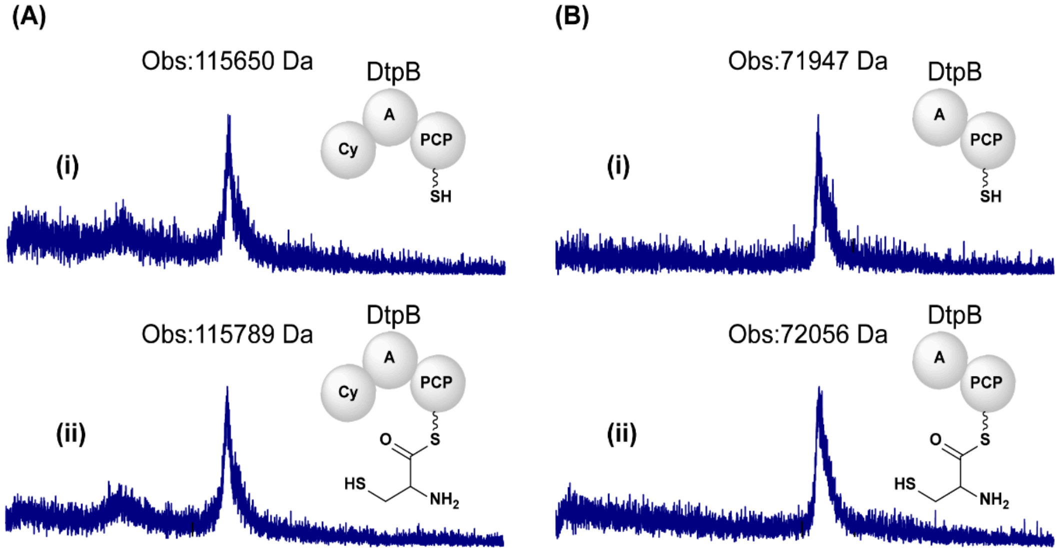

Figure S3. MALDI-TOF results of Cys loading assays of (A) DtpB and (B) DtpB-APCP.

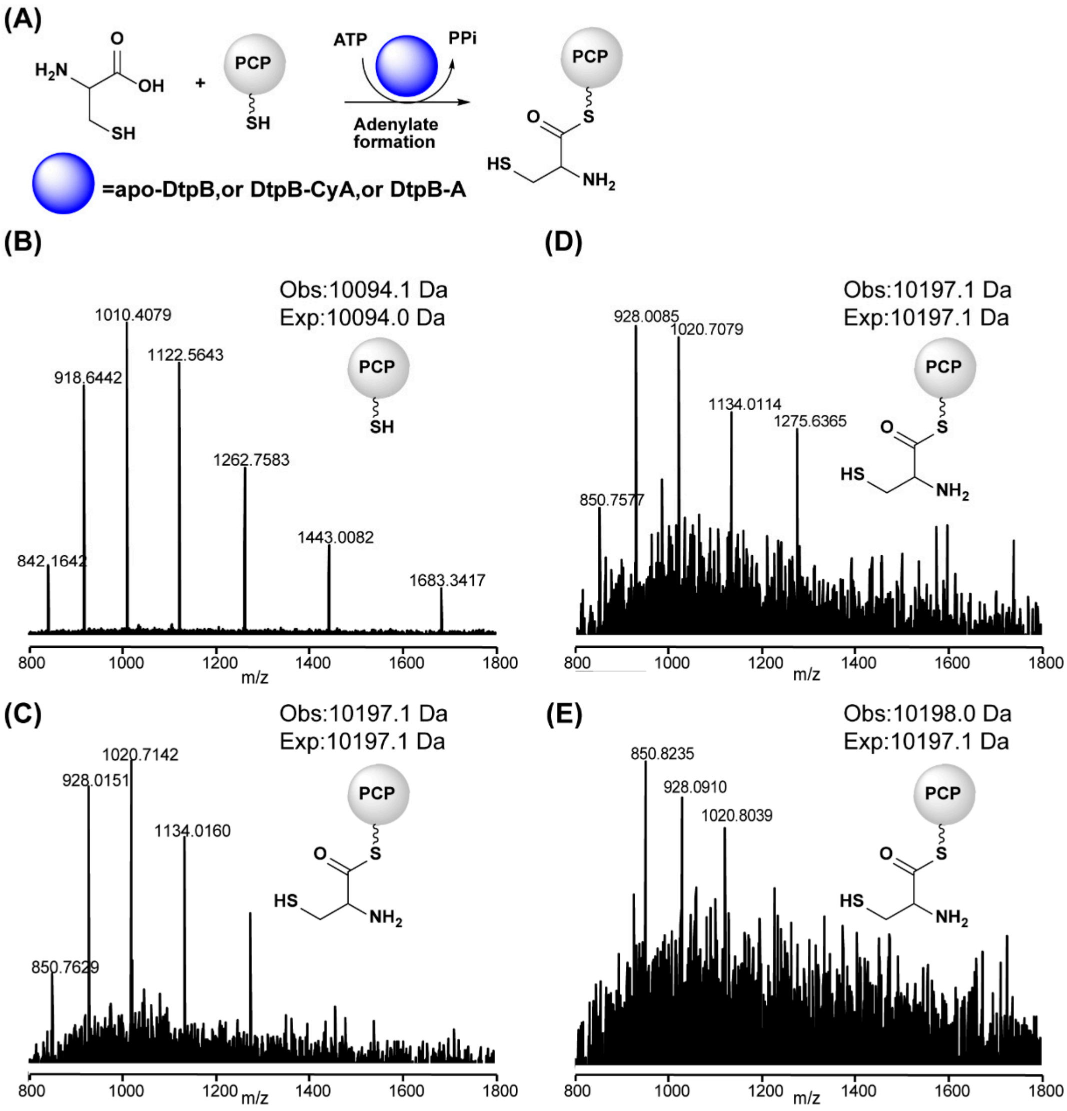

Figure S4. Reaction results of DtpB-PCP with adenylation domains and cysteine at the intermolecular stage. MS mass/charge (m/z) envelopes and mass deconvolution calculation (Obs.) and expected mass (Exp.) were shown. (A) Reaction diagram. (B) MS spectrum of product from reaction with only holo-DtpB-PCP. (C) MS spectrum of product from reaction with holo-DtpB-PCP and individual DtpB-A. (D) MS spectrum of product from reaction with holo-DtpB-PCP and apo-DtpB. (E) MS spectrum of product from reaction with holo-DtpB-PCP and DtpB-CyA bidomain.

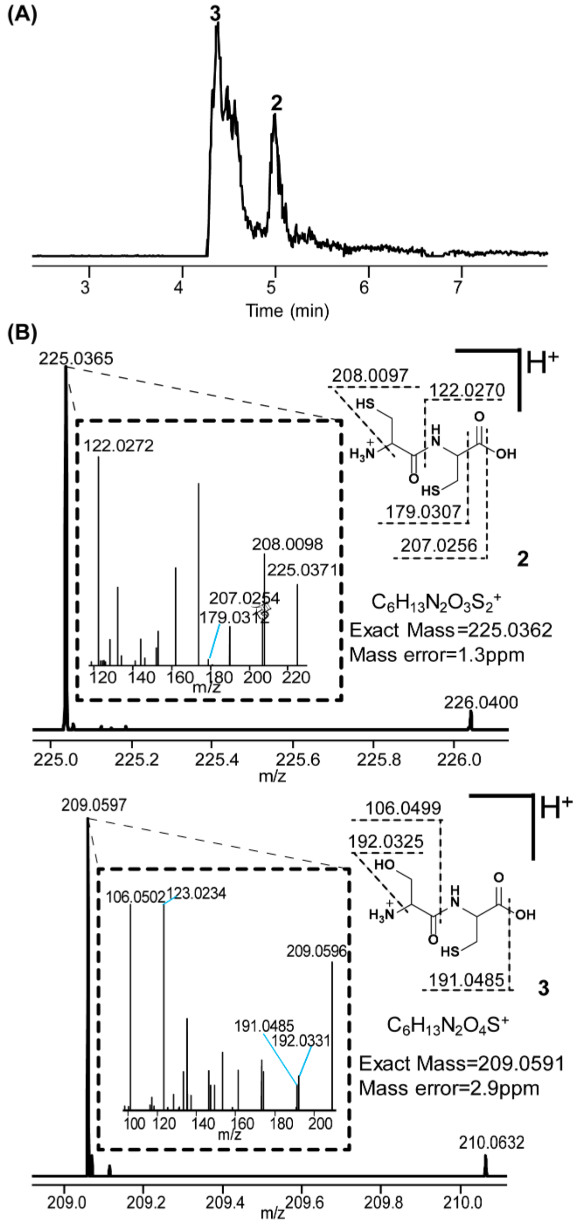

Figure S5. MS spectrums of dipeptides produced by individual A domain apo-DtpB. (A) Molecular weight extraction of TIC of the products of the reaction. (B) MS^2 spectrum of Cys-Cys dipeptide with observed MS=225.0371 and Ser-Cys dipeptide with observed MS=209.0597.

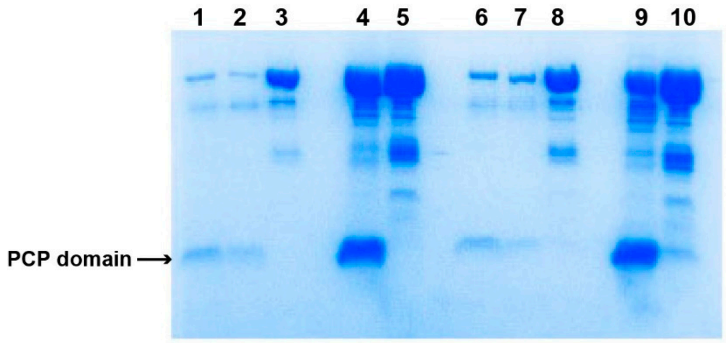

Figure S6. Tricine-SDS-PAGE of Ni-NTA separation of DtpB-APCP after incubation without cysteine and TEV digestion. (Line 1) FT of sample digested with TCEP. (Line 2) 25 mM imidazole washing out of sample digested with TCEP. (Line 3) 300 mM imidazole washing out of sample digested with TCEP. (Linea 4) concentrated mixture of FT and 25 mM imidazole washing out the ample digested with TCEP. (Line 5) concentrated 300 mM imidazole washing out of sample digested with TCEP. (Line 6) FT of sample digested without TCEP. (Line 7) 25 mM imidazole washing out of sample digested without TCEP. (Line 8) 300 mM imidazole washing out of sample digested without TCEP. (Linea 9) concentrated mixture of FT and 25 mM imidazole washing out of sample digested without TCEP. (Line 10) concentrated 300 mM imidazole washing out of sample digested without TCEP.

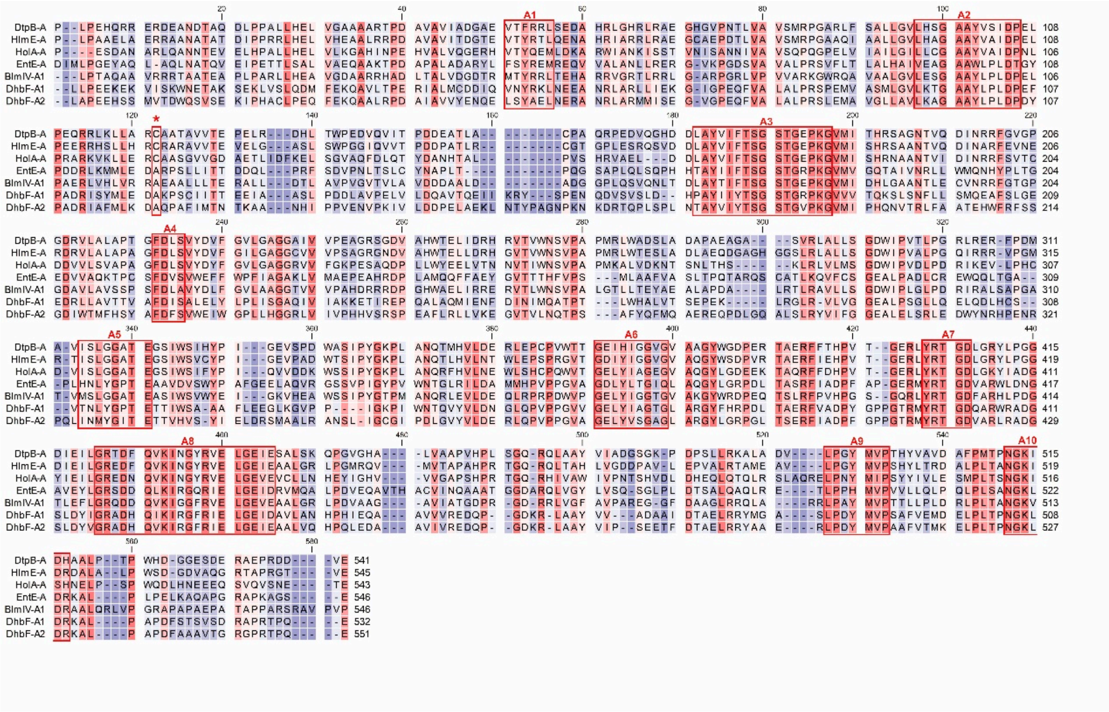

Figure S7. Multiple sequence alignment of adenylation domains. Sequences were aligned using CLC Genomics Workbench. 10 conserved regions A1-A10 are boxed with red lines. Conserved cysteine in A domains from dithiolopyrrolones is marked withed a red star sign.

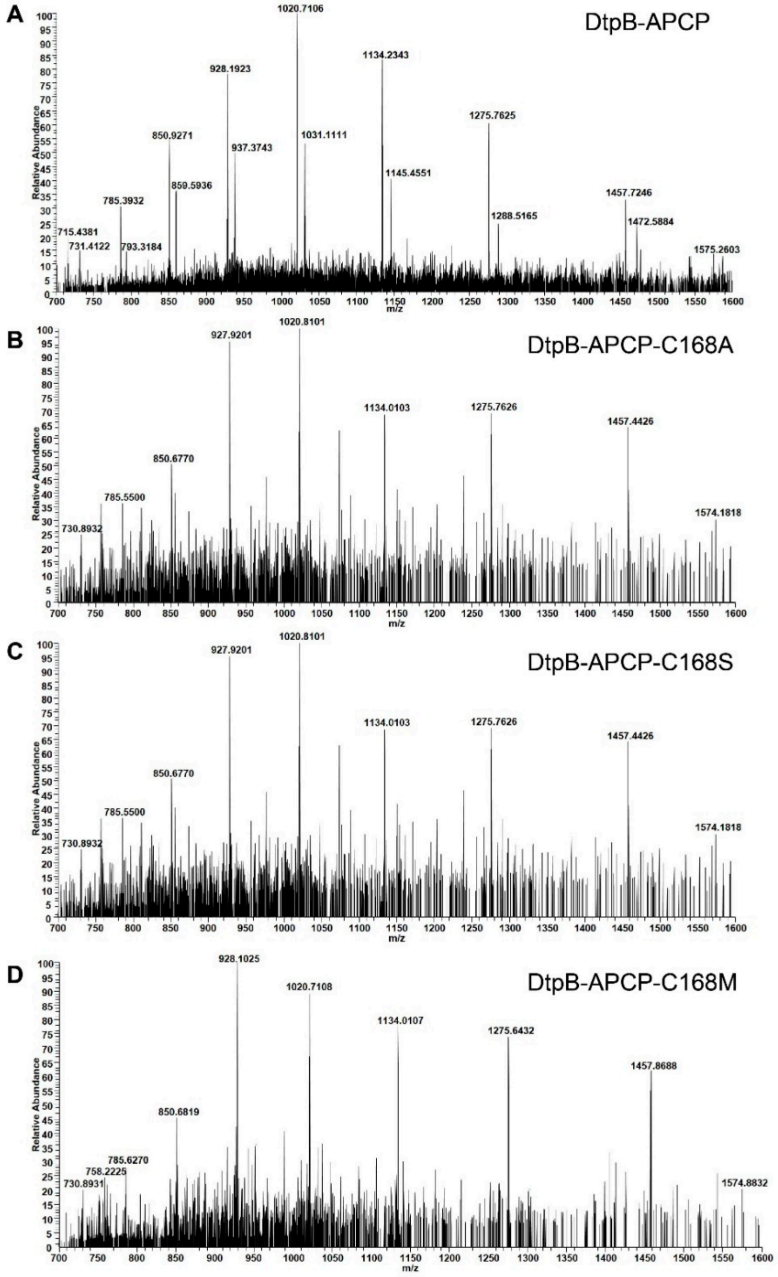

Figure S9. MS spectrum results of cysteine loading assays with site-mutated A-PCP bidomain of DtpB. (A) MS spectrum of extracts from assay using original APCP. Signals for single Cys loaded PCP and double Cys loaded PCP were shown clearly. (B) MS spectrum of extracts from assay using C168A mutated APCP. Only signals for single Cys loaded PCP can be discovered. (C) MS spectrum of extracts from assay using C168S mutated APCP. Only signals for single Cys loaded PCP can be found. (D) MS spectrum of extracts from assay using C168M mutated APCP. Only signals for single Cys loaded PCP can be found.

Table S1. PCR primers used for protein expression.

Primer	Sequence
DtpB-F	GTGCCGCGCGGCAGC<u>CATATG</u>GTGATCGGCCGCCACCGG
DtpB-R	GTGGTGGTGGTGGTG<u>CTCGAG</u>TCATGCGCGCACCTTCACCAG
DtpB-APCP-F	GTGCCGCGCGGCAGC<u>CATATG</u>GACATGTTCGACGCCTACC
CyA-TEV-F	GTTAATACAGATGTAGGTGTTCCACAGG
CyA-TEV-R	CCCG<u>GCCCTGAAAATACAGGTTTTC</u>CTCGTCGCTCTCGCCGCCGTC
TEV-PCP-F	CGAG<u>GAAAACCTGTATTTTCAGGG</u>CCGGGCCGAACCGCGCGACGAC
TEV-PCP-R	GTGGAACACCTACATCTGTATTAACGAAG

Table S2. HPLC elution profile for dipeptide.

Time (min)	A (V%)	B (V%)
0	95	5
3	95	5
13	80	20
15	10	90
17	10	90
18	95	5
20	95	5

Table S3. HPLC elution profile for PCP domain.

Time (min)	A (V%)	B (V%)
0	80	20
3	60	40
10	40	60
20	20	80
25	80	20
30	80	20

Table S4. PCR primers for Site-directed mutagenesis[a]

Primer name	Sequence（5’-3’）
DtpB/APCP-NdeI-F	TGGTGCCGCGCGGCAGCCATATG
DtpB/APCP-XhoI-R	TCTCAGTGGTGGTGGTGGTGGTGCTCGAG
C566A/C168A-F	AAGCTGCTGGCGCGCGCAGCCGCCACGGCCGTCG
C566A/C168A-R	ACGACGGCCGTGGCGGCTGCGCGCGCCAGCAGCTTC
C566S/C168S-F	AAGCTGCTGGCGCGCAGCGCCGCCACGGCCGTCG
C566S/C168S-R	ACGACGGCCGTGGCGGCGCTGCGCGCCAGCAGCTTC
C566M/C168M-F	AAGCTGCTGGCGCGCATGGCCGCCACGGCCGTCG
C566M/C168M-R	ACGACGGCCGTGGCGGCCATGCGCGCCAGCAGCTTC

[a]C: cystenine(Cys); A: alanine(Ala); S: serine(Ser); M:methionine(Met) .
